# When teaching procedures in simulation, do simulation adjuncts translate to better performance?

**DOI:** 10.1186/s41077-025-00365-z

**Published:** 2025-07-01

**Authors:** Jennifer Yee, Kimberly Bambach, David P. Way, Christopher E. San Miguel, Cynthia G. Leung, Scott Winfield, Rami A. Ahmed

**Affiliations:** 1 The Ohio State University Wexner Medical Center, Columbus, USA; 2 Indiana University, Bloomington, USA

**Keywords:** Education, Medical, And graduate; Emergency medicine; Hemorrhage, Esophageal, And gastric varices; Balloon tamponade

## Abstract

**Background:**

Learners should ideally be taught low-frequency, high-acuity procedures in a simulated clinical environment to limit patient harm. Evidence supporting a simulation scenario with educational adjuncts to teach procedures versus a traditional procedure laboratory have not been previously demonstrated. To investigate the effects of simulation adjuncts on procedural skills attainment, we compared performances of learners who trained on a modified airway task trainer within the context of a simulation scenario with educational adjuncts for balloon tamponade placement to those who trained on the same task trainer in a typical procedure laboratory setting.

**Methods:**

Fifty learners completed the curriculum: 37 emergency medicine residents, 8 emergency medicine/internal medicine residents, and 5 gastroenterology fellows. Learners were randomized into a simulation scenario with adjuncts (SA) or a control group using a modified task trainer in a procedure laboratory (PL) setting. We conducted baseline, approximately 1-month, and 5-month post-training assessments of self-identified competence, knowledge of the procedure, and observed procedural skills.

**Results:**

Learners from both groups demonstrated significant improvement on all three assessments from baseline to the first post-training session. Between the first and second follow-ups, both groups significantly improved on self-assessed competence. At the second follow-up, the PL-trained group scored significantly higher than the SA group on the performance assessment.

**Conclusions:**

All learners demonstrated significant improvements in knowledge, skills performance, and feelings of competence. The PL group demonstrated significantly higher skills performance during the second follow-up after training. This finding suggests that structured practice alone is an effective learning strategy for balloon tamponade placement without needing the resources of accompanying adjuncts within a simulation scenario, and that education with additional adjuncts may contribute to skills decay over time.

**Supplementary Information:**

The online version contains supplementary material available at 10.1186/s41077-025-00365-z.

## Introduction

Simulation is a proven method to acquiring and maintaining skills for procedures that are uncommonly encountered in the clinical environment. Learners may refine their skills through structured practice in a controlled and safe learning environment, where they are able to get the skills repetition needed without harming actual patients [1–3]. The fidelity of task trainers has been described in three different domains: equipment (the degree to which the simulator duplicates the appearance and feel of the actual system), environmental (the extent to which the simulator duplicates sensory information from the task environment), and psychological (the ability to suspend disbelief) [4].

Currently, it is unclear how similar a training situation must be to the actual task environment to provide effective training [5]. Traditionally, a positive correlation between simulation fidelity and transfer of training has been postulated [6]. It is hypothesized that simulation is most effective when it is performed in an environment that aims to mirror the actual setting, thereby ensuring that learners are able to suspend disbelief and perform as they would in the actual environment. In medical simulation, this includes “creating a learning environment that looks and feels like the real world… what is needed is an immersive environment that utilizes real medical equipment to make the simulation as life-like as possible” [2]. Skill retention is thought to be facilitated by simultaneously engaging multiple sensory systems within an immersive, realistic learning environment [7, 8]. High-fidelity simulators are created to help learners increase their visuospatial and perceptual skills and to sharpen their responses to critical incidents [2]. Issenberg et al. have suggested that simulator validity is contingent upon the degree to which the simulator approximates the scenario in which clinical care is delivered [3]. However, Beaubien and Baker challenge the assumption that educators should default to higher-fidelity methods, as previous studies have not demonstrated a direct correlation between simulation fidelity with training-related outcomes, including immediate learning, long-term transfer, and patient safety [4]. Furthermore, cognitive load theory may support the use of a simplified learning environment, as introducing additional distraction to the learning situation may exceed learners’ cognitive capabilities and ultimately inhibit learning [9].

When considering the best methods to ensure environmental fidelity in a nonclinical teaching environment, educators have used simulation spaces mocked up to mimic the clinical space, including supporting equipment such as crash carts, intravenous fluid poles, and cardiac monitors. While these methods are typically used for simulation case scenarios, the benefit of using a such simulation adjuncts to teach procedures has yet to be described. Therefore, we conducted a randomized controlled experiment between two procedural educational settings to determine the need for inclusion of simulation adjuncts: one that introduced additional environmental realism and one that reflected a traditional procedural laboratory setting with minimal physical resemblance to an actual clinical environment.

We chose to assess balloon tamponade device placement as this is an infrequent procedure in the clinical setting [10] and is usually performed under emergent, high-stress situations. The purpose of this project was to determine if trainees would better learn and retain the complex balloon tamponade placement procedure on a modified airway task trainer within a simulation scenario using environmental adjuncts (SA) such as auditory alarms and vital sign monitoring compared to trainees who learned and practiced on the same task trainer in a procedure laboratory (PL) setting.

## Methods

### Population and sample

We conducted this randomized comparison study at a quaternary-care university-affiliated teaching hospital during the second half of the 2020–2021 academic year. Learners included categorical emergency medicine (EM) residents, emergency medicine-internal medicine (EM-IM) residents, and gastroenterology (GI) fellows. These learners were selected as participants given the likelihood that they would be called upon to place balloon tamponade devices as part of their selected specialty. The study was deemed exempt by our institutional internal review board (IRB no. 2020E0236). STROBE reporting guidelines were followed to assemble this paper.

### Development of training assessments

#### Self-assessment for competence

We developed a 7-item survey for learners to self-assess their competence in the balloon tamponade procedure. The survey was designed to measure competence in the following: knowing the indications for a balloon tamponade device, gathering and preparing required equipment, placing and confirming placement of the device, troubleshooting device placement, and writing orders for device management. Learners rated their competence using a 5-point Likert-type scale, with one representing novice performance and five representing expert performance (see Appendix 1: Self-Assessed Competency Assessment).

#### Multiple-choice medical knowledge examination

A medical knowledge examination consisting of 15 multiple-choice items was jointly developed by 3 EM and 1 (GI) faculty member (see Appendix 2: Medical Knowledge Examination).

All three of the EM faculty regularly teach procedural skills to EM residents and serve as departmental educational faculty. Content included indications and contraindications for device placement, differences between types of balloon tamponade devices, procedural methodology, and post-placement management. The examination was administered as a baseline assessment prior to training, as a posttest at approximately 4 weeks after training, and as a follow-up test at approximately 5 months after training. Parallel forms of the examinations were created through randomization of the order in which items and options were presented.

#### Performance assessment

The procedural competency of the learners was assessed with a critical action checklist composed of 21 items developed through consensus and an iterative process of a panel of EM and GI faculty. The package inserts included with the balloon tamponade device were used as the primary reference in creating the checklist, as there are no published guidelines from any professional organization. The scenario required insufflation of both the gastric and esophageal balloons for hemorrhage control to be successfully achieved. Each item was scored dichotomously (yes/no). Rater training was performed to ensure understanding and appropriate use of the assessment tool. This checklist is listed in Appendix 3: Critical Actions.

### Procedure kit and preparation

Development of our modified airway task trainer is described elsewhere [11]. Each modified task trainer was accompanied by procedural equipment to perform the placement of the esophageal balloon tamponade device (balloon tamponade placement kits). The contents of these kits are listed in Appendix 4: Contents of the Procedure Kit.

A few basic preparatory steps were taken to optimize the available equipment. Stopcocks were placed into the sphygmomanometer tubing prior to the session to mitigate any confusion on how to attach stopcocks. The Kelly clamps had silk tape pre-wrapped around their ends to prevent damage to the esophageal balloon tamponade device. Facilitators had Magill forceps at their disposal to facilitate the passage of the device into the stomach, as the device frequently had difficulty advancing past the mid-esophagus without assistance.

### Teaching methods

We used structured practice to teach balloon tamponade placement to both the SA and PL groups. We used the 21-item critical action checklist so learners could engage in formative activities to achieve clear objectives.

We scheduled learners for each training session using an online sign-up form. As they signed up, learners were then randomly assigned to either the control PL group or the experimental SA group. A total of 27 learners were assigned to each study group which was stratified by education level and residency program.

Learners were not provided educational materials to study in advance of their baseline assessments. Four EM faculty members and one EM chief resident served as study facilitators based on availability. Facilitators divided responsibilities between pre- and post-testing and leading SA and PL sessions. The training syllabus implemented throughout the study is outlined in Appendix 5.

For pre-testing and formative education, learners were instructed to provide a unique non-identifiable four-digit code to de-identify their results. Learners then completed demographic forms, baseline self-assessed competency, and baseline medical knowledge assessments. Afterwards, the facilitator began the individual baseline skills assessments by verbally providing a case stem. One learner was assessed at a time in a simulation classroom setting by one faculty member. This stem included the acknowledgement that the patient had already been intubated, and that other healthcare providers were congruently actively performing supportive resuscitative measures such as transfusion of blood products. Learners were given 15 min to complete the procedure and were scored in real time using the predetermined critical actions checklist. Facilitators did not provide feedback on the learner’s performance during this stage.

**Fig. 1:**
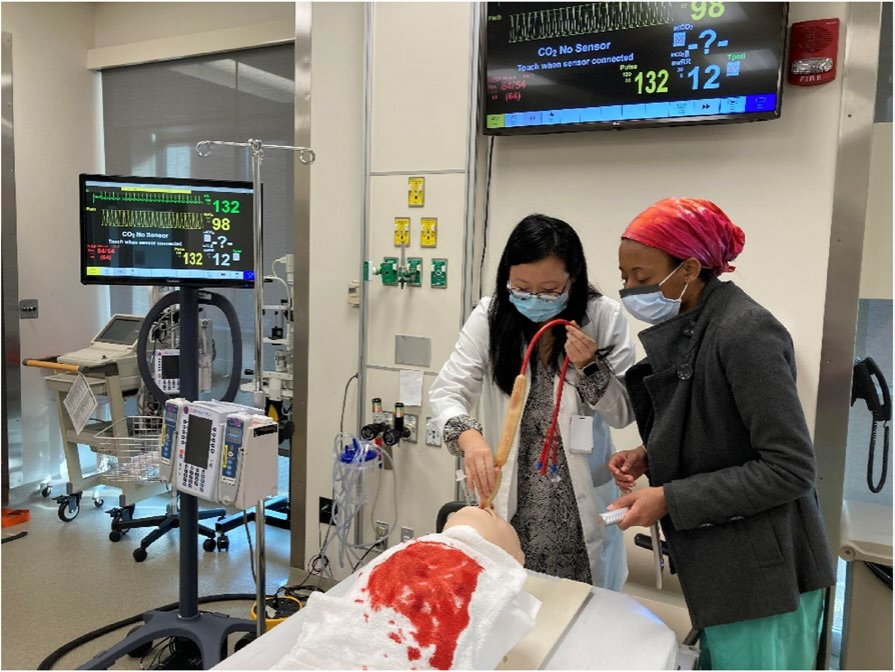
Simulation session with adjuncts session with two learners placing a balloon tamponade device

Once learners completed pre-testing, they were sent to their assigned group, either PL or SA, which consisted of two to four learners each. A template of teaching points was also distributed to faculty to ensure standardization of educational materials between both groups. Facilitators who led the SA group provided their learners with the same case stem used in the pre-testing session and then brought them into the simulation bay. The simulation bay was designed to resemble an acute resuscitation room in the emergency department, both aesthetically and with available equipment. The modified SA task trainer was hooked up to a cardiac monitor with auditory alarms noting the patient’s tachycardia. A towel with moulaged blood was placed on the patient’s chest. The same case stem from the checklist was provided at the beginning of the case to introduce the learners to the scenario (Appendix 3). Learners were encouraged to work together over a 15-min time period to place the esophageal balloon tamponade device (Fig. 1).

Vital signs remained fixed throughout the scenario. Afterwards, the facilitator reviewed the pre-determined learning points with the learners, including demonstrating the task from start to finish. Learners then took turns placing the device themselves while receiving real-time personalized feedback from facilitators. Subsequently, learners had 15 min for a simulation experience with 45 min of structured practice as led by the facilitator, leading to a total of 60 min for the SA instructional session.

**Fig. 2:**
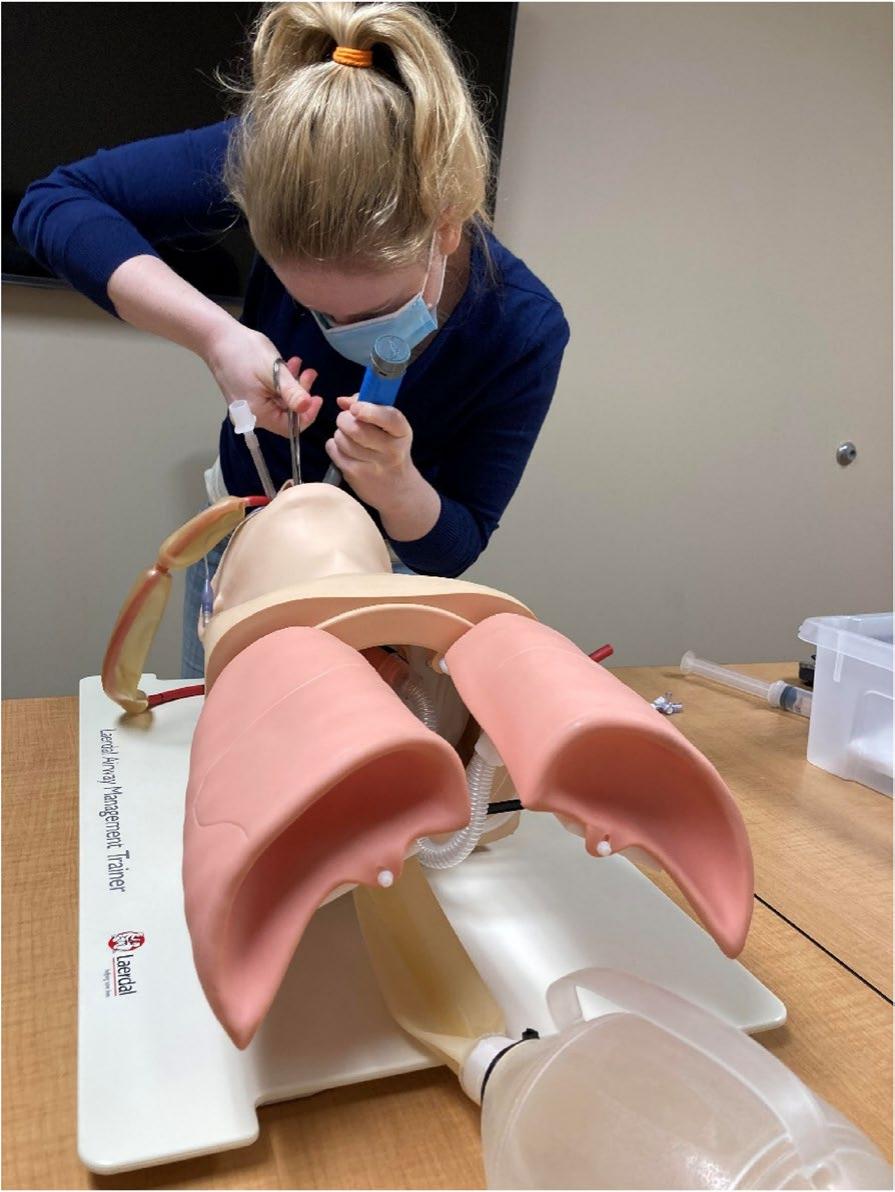
Procedural laboratory session with a learner placing a balloon tamponade device

The PL group assembled in a classroom that is typically used for teaching procedures. The modified airway task trainer and materials kit were placed on a classroom table. The PL facilitator reviewed the pre-determined learning points with learners, but did not provide a patient case stem. Facilitators demonstrated the task from beginning to end with the balloon tamponade device while highlighting critical actions. The learners then went through the process themselves (Fig. 2) while receiving structured feedback from the facilitators.

Ultimately, both the SA and PL groups were given the same 45-min time frame of education and skills practice. Learners from both groups were expected to practice the entire procedure two times during their assigned session.

Learners were reassessed twice more, each time on an individual basis and in the same simulation classroom setting, approximately 1 month (first posttest) and then 5 months (second posttest) after the initial education intervention session. Learners repeated a self-assessment of competency and medical knowledge exam, followed by an observation of performance by one faculty member against the same critical action checklist. Each reassessment session took about 35 min. After each reassessment, facilitators provided learners with focused feedback to review which critical actions were not yet achieved.

### Statistical analysis

We conducted a preliminary power analysis for Global Effects multivariate analysis of variance (MANOVA) to estimate the number of subjects needed for this study (G*Power 3.1). We estimated that the effect size of a direct educational intervention would be moderate to large (0.80), thus making it easier to detect; consequently, we used these input parameters [effect size = 0.80 (large); *α*-error probability = 0.05; power = 0.95, groups = 2, and number of measurements = 3]. The total sample size needed for detecting a large effect was *N* = 26 or 13 per group.

**Fig. 3:**
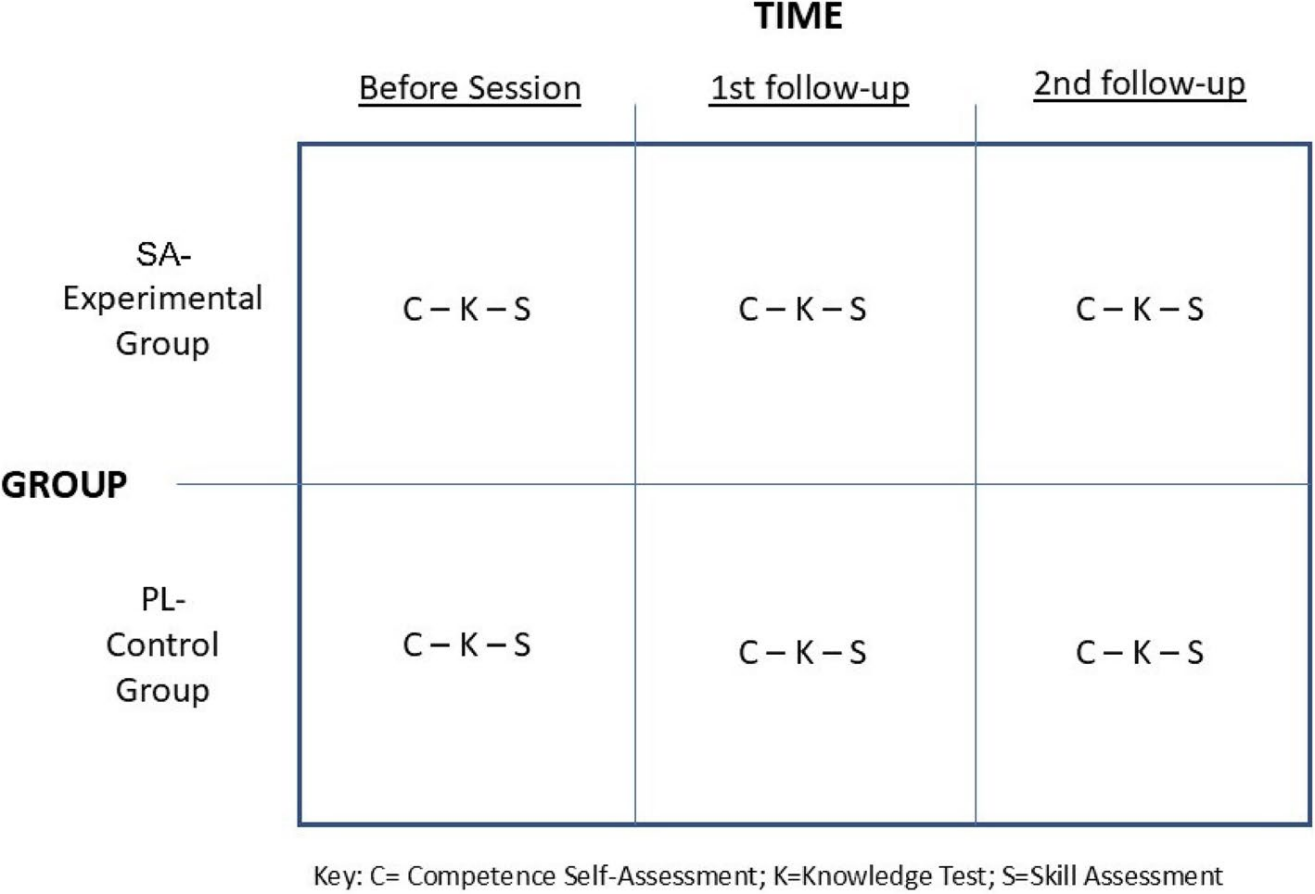
Two-way (2 × 3) multivariate analysis of variance (MANOVA) study design diagram. C, competence self-assessment; K, knowledge test; S, skill assessment; SA, simulation scenario with adjuncts; PL, procedure laboratory. Note: The first follow-up was at least 12 days after the session but could have been as long as 104 days. The second follow-up was at least 66 days after the session but could have been as long as 183 days

We compared the two groups (the SA experimental and PL control groups) using a two-way (2 × 3) multivariate analysis of variance (MANOVA), with one repeated measure (Fig. 3). We depicted a group by time design with three dependent measures: competence self-assessment, knowledge, and skills.

The intervention groups and time of assessment were the independent variables, and the three measures served as the dependent variables. The Global Effects MANOVA helps to preserve power by limiting the number of paired comparisons required. Only significant interactions and main effects were analyzed with post hoc tests. Statistical analyses were performed with IBM-SPSS Version 28 (IBM Corp. released 2021, IBM SPSS Statistics for Windows, Version 28.0, Armonk, NY, USA: IBM Corp.).

## Results

### Demographics

Our institution has 63 EM and EM/IM residents and 14 GI fellows. We gathered complete study data from 50 trainees, including 71.43% (45 of 63) of EM and EM/IM residents and 35.71% (5 of 14) of GI fellows. Study participants’ demographics are shown in Table 1.. All trainees completed all three assessments other than three EM PGY-3 residents in the SA group (one performed the first two assessments, and two only performed the baseline assessment) and one GI PGY-4 fellow in the PL group (performed the first two assessments only).

**Table 1:** Frequencies and percentages of study participants by specialty, level of training, gender, and time between assessments

		HF experimental (*N* = *24*)	PL control (*N* = *26*)	Total (*N* = *50*)
Program	EM	18 (77.8)	19 (70.4)	37 (74.0)
	EM/IM	2 (7.4)	6 (22.2)	8 (14.8)
	GI	4 (14.8)	1 (7.4)	5 (10.0)
	Total	24 (100)	26 (100)	50 (100)
Level of training	PGY-1	7 (25.9)	10 (37.0)	17 (31.5)
	PGY-2	8 (29.6)	6 (22.2)	14 (25.9)
	PGY-3	4 (25.9)	8 (29.6)	12 (24.0)
	PGY-4	3 (11.1)	1 (7.4)	4 (8.0)
	PGY-5	2 (7.4)	0	2 (3.7)
	PGY-6	0	1 (3.7)	1 (1.9)
	Total	24 (100)	26 (100)	50 (100)
Age (years)				
	Mean (SD)	29.58 (2.55)	30.38 (3.40)	
Time between assessments (days)	Mean (SD)			
	Baseline to 1 month	Mean 48.79 (20.59) (15–104 days)	Mean 46.54 (21.78) (12–102 days)	
	1 to 5 months	Mean 130.46 (35.39) (66–178 days)	Mean 119.17 (34.45) (66–183 days)	

*SA* simulation scenario with adjuncts,

*PL* procedure laboratory,

*EM* emergency medicine,

*IM* internal medicine,

*GI* gastroenterology,

*PGY* postgraduate year,

*SD* standard deviation

Three members of each group reported prior experience with the placement of balloon tamponade devices. In the SA group, two had experience in a procedural laboratory, and one had clinical experience. In the PL group, all three learners had mixed experiences in the procedural laboratory, a cadaver lab, and in a simulation session. No one in the PL group had clinical experience.

**Fig. 4:**
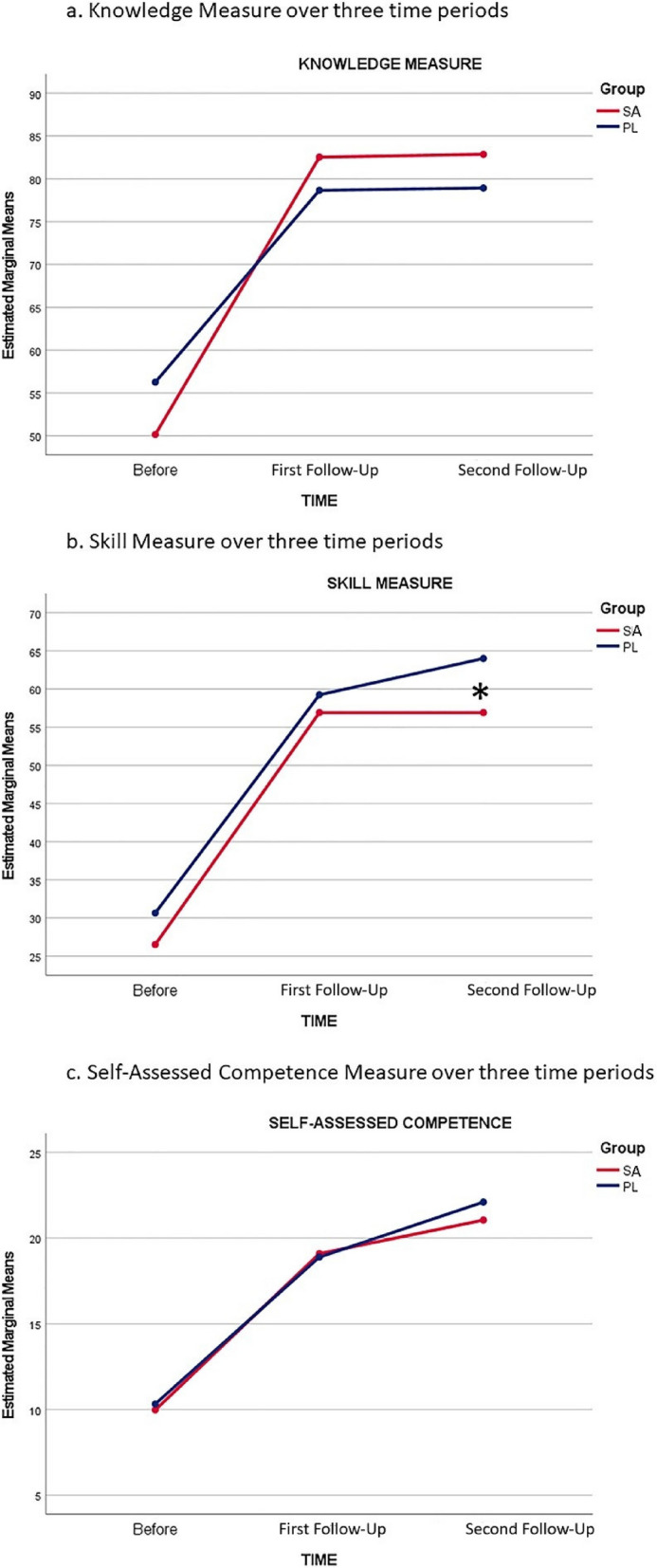
Knowledge, skill, and self-assessed competency measures over three time periods. SA, simulation scenario with adjuncts; PL, procedure laboratory. *The PL control group scored significantly higher than the SA intervention group on the skill measure at the second follow-up

The global omnibus MANOVA resulted in a significant main effect for time (*F* = 55.2; *df* = 6, 39; *p* ≤ 001; *es* = 0.90), but not for group (*F* = 1.8, *df* = 3, 42; *p* = 0.163; *es* = 0.114). The partial eta effect sizes for the time effect are considered large [12]. Post hoc tests and estimated means plots (see Fig. 4a, b, c) of the Time factor showed that all three dependent measures were statistically significant over the three time points (before, first follow-up, and second follow-up). The resulting *F*-tests with the Huynh–Feldt correction for violation of the assumption of sphericity were as follows:

- $$\mathrm{Knowledge}\;(F\;=\;123.3;\;df\;=\;1.5,\;65;\;p\;<\;001;\;es\;=\;0.74)$$ 
- $$\mathrm{Skill}\;(F\;=\;106.4;\;df\;=\;1.8,\;65;\;p\;<\;001;\;es\;=\;0.71)$$ 
- $$\mathrm{Self}\;(F\;=\;202.0;\;df\;=\;1.9,\;65;\;p\;<\;001;\;es\;=\;0.82)$$ 

Inspection of the three estimated mean’s plots makes it clear that the source of statistical significance related to the Time factor, for all three dependent measures, was the increase in scores from before to after the training (Time 1 to Time 2). There is also a source of statistical significance related to the Group factor by looking at the difference in scores on the skill measure or performance assessment. Here, we see that the PL control group scored significantly higher than the SA intervention group on the second follow-up assessment (see * on Fig. 4b).

Learners from both groups scored higher on the medical knowledge with balloon tamponade device placement between baseline assessments and first follow-up after instruction. Both groups seemed to sustain that knowledge with comparable scores on the knowledge test at the second follow-up (Fig. 4a). Learners’ self-assessment of their own competence in placing the balloon tamponade device also increased significantly as measured at baseline and the first follow-up after instruction. But again, both groups sustained their self-assessed competence at the second follow-up (Fig. 4c).

## Discussion

Learners demonstrated improved self-assessed competency, medical knowledge, and procedural performance in placing balloon tamponade devices after structured practice in either a simulation scenario using additional adjuncts or a traditional procedural laboratory setting. There were no significant differences in procedural performance between these educational settings, suggesting that the inclusion of simulation adjuncts in procedural simulation training is not as important as previously thought.

Historically, the structural fidelity of the task trainer, including physical resemblance and environmental adjuncts, has been assumed to increase learner engagement, thereby enhancing transfer of knowledge. However, interpretation of historical data may have been hampered by variability in the definition of fidelity as it pertains to simulation [13]. Previous studies largely focused on the fidelity of equipment used in surgical procedures [14–18] and lacked robust evidence comparing learners’ procedural skills before and after training [19]. More recent reports suggest that procedural training with low-fidelity models is not inferior to high-fidelity models, and that high-fidelity simulators are not associated with better procedural skills transfer [13]. As reviewed by Hamstra et al., an increasing body of evidence suggests that there are complex interactions between the simulator, the learner, and the instructors that determine the effectiveness of educational simulation, and that “the functional alignment with the learning task, the instructional design, and the instructor likely have far greater impact on immediate learning, retention, and transfer to new settings” [20]. Our findings lend further support to this concept.

After observing a significant increase in scores from Time 1 to Time 2 for measures of knowledge, skill, and self-assessed competence in both groups, we observed no statistically significant changes from Time 2 to Time 3 — except for a significant improvement in the PL group’s score in skill performance at Time 3 relative to the SA group’s performance. Interestingly, neither group exhibited significant skill decay between the first and second follow-up testing sessions. Rather, the observed difference in performance was driven by continued improvement in the PL group. The mean latency to testing at the second follow-up was slightly shorter for the PL group (119 days) compared to the SA group (130 days). Possibly, we may have inadvertently caught the learners at a critical point in the skills decay curve. If the peak of skill acquisition was between our first and second testing intervals, we could be observing the beginning of skill decay in both groups. However, we think this is unlikely as it would run counter to the body of evidence which demonstrates that performance on rarely performed tasks peaks shortly after intensive training [21]. Perhaps because the testing environment more closely resembled the practice environment for the PL group, the first testing session served more of a role as continued practice than it did for the SA group. Regardless of the cause, the observed difference between groups was modest and is unlikely to be clinically impactful. As discussed above, the more substantial finding is that structured practice, common to both testing groups, resulted in substantial skill acquisition without evidence of significant skill decay at 4 months.

We hypothesize that perhaps the adjuncts used in the SA group may have contributed to longer-term skills decay. Our findings are congruent with cognitive load theory, which suggests that minimizing cognitive load by simplifying the learning environment may allow for more efficient learning [9]. Literature on the relationship between structured practice and procedural skill decay in the simulation setting is currently limited, so further studies are needed to investigate this finding.

### Limitations

This was a small pilot cohort conducted at one institution. The self-assessed competence survey, medical knowledge test, and critical actions checklist were not previously validated in the literature, and there were no existing evidence-based guidelines for scaffolding. Instead, we created these tools based on content expertise. We recognize the limitations of learner self-assessment [22]; rather than measuring self-assessment in isolation, we wished to see how this variable was affected in comparison to medical knowledge and procedural skill.

There was some variability of timing with regard to the first and second posttests, as learners signed up depending on their availability of their schedules and may have affected statistical analysis as time points were treated as fixed. Group sizes were not standardized given variability in schedule availability; therefore, the amount of time committed to hands-on practice differed slightly between groups. We were limited in the amount of faculty raters, so we were unable to have more than one faculty member perform real-time assessments of learners to calculate inter-rater reliability. Raters were not blinded to the participants, and faculty may have been familiar with the participants, introducing a source of potential bias. True deliberate practice was not able to be performed to ensure mastery of all steps as there was a set amount of time learners were scheduled in the simulation laboratory and able to be away from clinical duties. Despite this limitation, we were able to demonstrate that even limited structured practice was effective.

The modified task trainer was cost-effective and well-suited to practice balloon tamponade device placement; however, we have not gathered evidence of validity for the use of this device. The task trainer was prone to difficulties such as advancing the balloon device down the esophagus into the stomach, requiring the facilitator to help pull the distal end of the device through the stomach using McGill forceps. The task trainer also had limited fidelity as learners were able to visualize the depth of the balloon prior to radiographic confirmation of placement. Other modified task trainers for balloon tamponade device placement have been described, each with their own benefits and compromises [23–25].

### Future directions

This study is the first to compare procedural performance for balloon tamponade device placement on a modified task trainer in a simulation-based scenario using environmental adjuncts with performance in a traditional procedural laboratory setting. Future studies may assess performance of other procedures in different learning environments. In addition, future studies are needed to determine effects of learning and testing environments on procedural skill decay in the simulation setting.

## Conclusion

An educational intervention with a cost-effective modified airway task trainer led to statistically significant improvement in procedural performance, medical knowledge, and self-assessed competency in placing balloon tamponade devices in both a traditional procedural laboratory setting and within a simulation scenario using environmental adjuncts. Use of environmental adjuncts in a simulation scenario did not provide any additional benefit to learners on their measured performance.

## Supplementary Information

Supplementary Material 1. Appendix 1: Self-Rated Competence Survey.

Supplementary Material 2. Appendix 2: Medical Knowledge Exam.

Supplementary Material 3. Appendix 3: Critical Actions.

Supplementary Material 4. Appendix 4: Contents of the Procedure Kit.

Supplementary Material 5. Appendix 5. Balloon tamponade device placement training sessions.

## Data Availability

No datasets were generated or analysed during the current study.
