# Supplementary material for: When teaching procedures in simulation, do simulation adjuncts translate to better performance?
Source: Adv Simul (Lond). 2025 Jul 1;10:36. doi: 10.1186/s41077-025-00365-z (PMC12219805; doi:10.1186/s41077-025-00365-z)
Supplement: Supplementary file 1 — Supplementary Material 1. Appendix 1: Self-Rated Competence Survey. [file 41077_2025_365_MOESM1_ESM.doc]

Appendix 1: Self-Rated Competence Survey

Please self-reflect about each skill and provide an honest assessment of your own level of competence at this point in time by selecting the rating that best fits. Use the following scale:

1= Very Unskilled (Novice, need constant supervision in executing this skill)

2= Unskilled (Beginning proficiency, but still need much guidance)

3= Intermediate Performer (Can do myself, with occasional guidance from an expert)

4= Skilled (Can perform fairly autonomously with little help from an expert)

5= Very Skilled (Expert, can teach and supervise others on this skill)

1. Assessing the need for a balloon tamponade device (such as a Blakemore or Minnesota tube)?
2. Gathering equipment needed to place a balloon tamponade device?
3. Preparing the equipment needed to place a balloon tamponade device?
4. Placing a balloon tamponade device?
5. Confirming placement of a balloon tamponade device?
6. Troubleshooting placement of a balloon tamponade device?
7. Writing orders for the management of a balloon tamponade device, once the device has been placed?
