# Supplementary material for: When teaching procedures in simulation, do simulation adjuncts translate to better performance?
Source: Adv Simul (Lond). 2025 Jul 1;10:36. doi: 10.1186/s41077-025-00365-z (PMC12219805; doi:10.1186/s41077-025-00365-z)
Supplement: Supplementary file 2 — Supplementary Material 2. Appendix 2: Medical Knowledge Exam. [file 41077_2025_365_MOESM2_ESM.doc]

Appendix 2: Medical Knowledge Exam

For brevity, “tube” will represent both Sengstaken-Blakemore and Minnesota tubes, unless clarified as such.

1. What is the difference between Sengstaken-Blakemore and Minnesota tubes?
2. The Minnesota tube has an esophageal and a gastric balloon, while the Sengstaken-Blakemore has only a gastric balloon
3. The Minnesota tube has an esophageal suction port

c. The Sengstaken-Blakemore tube has an esophageal suction port

d. The Sengstaken-Blakemore tube has an esophageal and a gastric balloon, while the

Minnesota has only a gastric balloon

e. The tubes are the same in structure and differ in brand name alone

1. Which statement is accurate regarding gastric balloon size?
2. The Minnesota tube has a smaller balloon (250 mL)
3. The Minnesota tube has a larger balloon (450 mL)

c. The Sengstaken-Blakemore tube has a larger balloon (450 mL)

d. The Sengstaken-Blakemore has a smaller balloon (100cc)

e. Both tubes have the same gastric balloon size (250 mL)

1. What is the purpose of tube placement?
2. Definitive management for any acute upper GI bleed

b. Definitive management for acute variceal hemorrhage

c. Temporizing measure for any acute upper GI bleed

d. Temporizing measure for acute variceal hemorrhage

e. To assess the source of bleeding

1. What is the suggested depth for tube placement, as measured from the incisors?
2. 30 cm
3. 35 cm

c. 40 cm

d. 45 cm

e. 50 cm

1. What is unnecessary when preparing to place the tube?
2. Checking balloons for leaks
3. Putting on personal protective equipment

c. Obtaining all equipment in advance

d. Stopping blood transfusion during the procedure

e. Tube and balloon lubrication

1. Which balloon should be inflated first?
2. Esophageal balloon

b. Gastric balloon

c. Both balloons should be inflated simultaneously

d. Either balloon may be inflated first

1. How do you confirm placement of the gastric balloon?
2. Inflate the gastric balloon with 50cc-100cc of air, then confirm placement with chest x-ray

b. Inflate the gastric balloon with 50cc-100cc of air, then confirm placement with KUB

c. Inflate the gastric balloon with 50cc-100cc of air, then aspirate, looking for gastric contents

d. If tube is advanced easily to desired length, auscultate over stomach. If air is heard, may inflate the gastric balloon with 50-100cc of air

e. If tube is advanced easily to desired length, may inflate to full volume, as easy advancement indicates tube is in the correct location

1. What is the final desired location of the gastric balloon?
2. Adjacent to the pylorus
3. Anterior wall

c. Any location in the stomach

d. In the fundus of the stomach

e. Pulled against the gastroesophageal junction

1. What is the next best step if the patient has ongoing bleeding despite gastric balloon inflation?
2. Add more traction to the tube
3. Administer more packed red blood cells

c. Continue aspirating from the gastric aspiration port

d. Inflate the gastric balloon until bleeding ceases

e. Inflate the esophageal balloon

1. What is the maximum pressure for esophageal balloon inflation?
2. 45 mmHg

b. 50 mmHg

c. 55 mmHg

d. 60 mmHg

e. Until bleeding is controlled

1. What must be done prior to tube placement?
2. Administration of intravenous fluids until hypotension is resolved
3. Endoscopy to confirm presence of varices

c. Intubation

d. Nothing must be done, as this is an emergent procedure

e. Reviewing the initial hemoglobin and platelet levels

1. How long may the tube be left in place?
2. Up to 6 hours
3. 6-12 hours

c. 24-36 hours

d. 72-96 hours

e. As long as it takes until patient undergoes endoscopy or TIPS

1. What is not a potential complication of tube placement?
2. Asphyxiation from tracheal compression
3. Aspiration

c. Esophageal perforation

d. Esophageal necrosis

e. SVC syndrome

1. What is a definitive contraindication to tube placement?

a. Anticoagulation use

b. Bleeding has temporarily ceased

c. INR > 1.2

d. Sustained hypotension

e. Thrombocytopenia < 100/mm³

1. What is not an appropriate way to secure the tube?
2. Commercial endotracheal tube securing device
3. Secure the tube to a football helmet face mask

c. Tie the tube to a length of gauze roll, hang the gauze over an IV pole, and tie a 500 mL bag of intravenous fluid to the other end to apply 0.5 kilograms of weight

d. Tie the tube to a length of gauze roll, then tie the gauze to a metal intravenous pole if connected to the bedframe

Appendix 2 Legend:

mL = milliliter

cm = centimeter

cc = cubic centimeter

KUB = kidneys ureter bladder

mmHg = millimeters of mercury

SVC = superior vena cava

INR = international normalized ratio
