# Supplementary material for: When teaching procedures in simulation, do simulation adjuncts translate to better performance?
Source: Adv Simul (Lond). 2025 Jul 1;10:36. doi: 10.1186/s41077-025-00365-z (PMC12219805; doi:10.1186/s41077-025-00365-z)
Supplement: Supplementary file 3 — Supplementary Material 3. Appendix 3: Critical Actions. [file 41077_2025_365_MOESM3_ESM.doc]

Appendix 3: Critical Actions

Case Scenario: 50-year-old man with a history of alcoholic cirrhosis presents to the emergency department by EMS with a chief complaint of “throwing up blood”. His initial vitals are as follows: BP 60/palp, HR 140, Resp 24, Temp 98°, O2 saturation 97% on room air.

Bloodwork was immediately drawn during triage. He was given Rocephin, an octreotide bolus with infusion, pantoprazole bolus with infusion, and two units of blood via rapid transfuser, but has had 1L of witnessed dark red hematemesis.

His vitals after rapid blood transfusion are as follows: 70/50, HR 130, Resp 24, Temp 98°, and O2 saturation of 96% on room air. He remains pale with thready pulses and cool extremities. His hemoglobin is 7.0 grams/deciliter, down from a baseline of 9.5 grams/deciliter, with a baseline thrombocytopenia of 80. Their international normalized ratio (INR) is 1.6.

You have been called for further management. Endoscopy is not available. The patient has just been intubated via direct laryngoscopy with endotracheal tube placement confirmed by chest x-ray.

1. Document emergency consent
2. Test both balloons for leaks by inflation with air
3. Remove air and clamp ports
4. Coat tube with lubricant
5. Elevate the head of the bed 30-45°
6. Insert the tube to 50 centimeters of depth (if the provider does not verbalize this out loud, observers should ask the tube depth after placement)
7. Auscultate over the stomach as 50 milliliters of air is injected into the gastric balloon
8. Obtain a chest x-ray to confirm gastric balloon placement in the stomach
   1. Note: NO CREDIT for a kidneys-ureter-bladder (KUB) film if a chest x-ray is not ordered
9. Correctly identify placement of the gastric balloon, including whether it is placed correctly or incorrectly
10. PROMPT: If the tube is placed incorrectly, what would you do? (deflate the gastric balloon, reposition the tube, AND order a repeat chest x-ray)
11. Inflate the gastric balloon with the recommended amount of air (250 cubic centimeters for Blakemore, 450 cubic centimeters for Minnesota)
12. Apply gentle traction until resistance is felt, signifying abutment against the gastroesophageal junction
13. Place the tube under traction with use of a football helmet or endotracheal tube holder or roller bandage/500 milliliter intravenous fluid bag hung over an intravenous pole
14. Attach the gastric lavage port to suction
15. Irrigate the gastric aspiration tube with 50 cubic centimeters of water to prevent clotted blood from plugging the tube
16. Reassess the patient’s vital signs and ask about how much blood has been suctioned.
    1. Note: if providers do not verbalize any action after suctioning, observers may ask “What would you do next?”
       1. Learners are told there is continued bleeding 30 minutes later despite lavage and suctioning
17. Connect manometer
18. Inflate the esophageal balloon to the lowest pressure needed to stop bleeding (facilitators will verbalize bleeding stops at 45 millimeters of mercury)
19. (Blakemore) clamp gastric suction port with Kelly clamps and apply continuous suction to the orogastric tube

(Minnesota) clamp the gastric suction port with Kelly clamps and apply continuous suction to the esophageal suction port

1. Obtain second chest x-ray to confirm placement
2. Write orders for esophageal balloon care (need all)
   1. Checking/documenting device depth, balloon pressure(s), characteristics and volume of drainage every hour
   2. Checking/documenting level of suction every two hours)
