# Supplementary material for: When teaching procedures in simulation, do simulation adjuncts translate to better performance?
Source: Adv Simul (Lond). 2025 Jul 1;10:36. doi: 10.1186/s41077-025-00365-z (PMC12219805; doi:10.1186/s41077-025-00365-z)
Supplement: Supplementary file 4 — Supplementary Material 4. Appendix 4: Contents of the Procedure Kit. [file 41077_2025_365_MOESM4_ESM.docx]

| Appendix 4: Contents of the Procedure Kit |
| --- |
|  |
| One intubating stylet |
| One 7-0 endotracheal tube |
| One endotracheal tube holder |
| One laryngoscope handle with a miller blade |
| One set of magill forceps |
| Two 60 milliliter catheter tip syringes |
| One 60 milliliter luer lock syringe |
| Two small catheter adapter connections |
| Two large multipurpose tubing adapters |
| One roll of silk tape |
| One roll of kerlix gauze |
| Two 3-way stopcocks |
| One 500 milliliter bag of normal saline |
| One 250 milliliter bottle of sterile water |
| One permanent marker |
| One lubricant spray bottle |
| Two packets of water-soluble lubricant |
| One sphygmomanometer |
| One straight Kelly forceps |
| One curved Kelly forceps |
| One stethoscope |
| One set of suction tubing |
| One Salem Sump™ nasogastric tube |
| One Minnesota tube |
| Three printed chest x-ray images of various balloon inflation amounts and locations |
