# Supplementary material for: When teaching procedures in simulation, do simulation adjuncts translate to better performance?
Source: Adv Simul (Lond). 2025 Jul 1;10:36. doi: 10.1186/s41077-025-00365-z (PMC12219805; doi:10.1186/s41077-025-00365-z)
Supplement: Supplementary file 5 — Supplementary Material 5. Appendix 5. Balloon tamponade device placement training sessions. [file 41077_2025_365_MOESM5_ESM.docx]

Appendix 5. Balloon tamponade device placement training sessions

| *Control Group*  *Baseline assessment and education* (Pre-testing and educational intervention) – 80 minutes per learner  Pre-test   - Pre-test self-assessment competency survey (5 minutes) - Pre-test multiple-choice exam (15 minutes) - Individual assessment on task trainer utilizing the critical actions checklist (15 minutes)   Educational Intervention   - Procedure Laboratory (45 minutes education and deliberate practice) | *Experiential Group*  *Baseline assessment and education* – 80 minutes per learner  Pre-test   - Pre-test self-assessment competency survey (5 minutes) - Pre-test multiple-choice exam (15 minutes) - Individual assessment on task trainer utilizing the critical actions checklist (15 minutes)   Educational Intervention   - High-Fidelity simulation scenario (15-minute simulation scenario, then 45 minutes education and deliberate practice) |
| --- | --- |
| *Approximately one month post-test* – 35 minutes per learner  Post-test #1   - Post-test self-assessment competency survey (5 minutes) - Post-test multiple-choice exam (15 minutes) - Individual assessment on task trainer utilizing the critical actions checklist (15 minutes) | *Approximately one month post-test* - 35 minutes per learner  Post-test #1   - Post-test self-assessment competency survey (5 minutes) - Post-test multiple-choice exam (15 minutes) - Individual assessment on task trainer utilizing the critical actions checklist (15 minutes) |
| *Approximately five months post-test* – 35 minutes per learner  Follow-up test #2   - Post-test self-assessment competency survey (5 minutes) - Post-test multiple-choice examination (15 minutes) - Individual assessment on task trainer utilizing the critical actions checklist (15 minutes) | *Day 3* *Approximately five months post-test* – 35 minutes per learner  Follow-up test #2   - Post-test self-assessment competency survey (5 minutes) - Post-test multiple-choice examination (15 minutes)   Individual assessment on task trainer utilizing the critical actions checklist (15 minutes) |
